# Genetic organization of an M protein trans-acting positive regulator (Mga) orthologue and its adjacent M-like protein (SCM) alleles in *Streptococcus canis*

**DOI:** 10.1186/s13104-024-06795-8

**Published:** 2024-05-15

**Authors:** Takashi Takahashi, Takahiro Maeda, Haruno Yoshida, Mieko Goto, Yuzo Tsuyuki, Jae-Seok Kim

**Affiliations:** 1 Laboratory of Infectious Diseases, Graduate School of Infection Control Sciences and Ōmura Satoshi Memorial Institute, Kitasato University, 5-9-1 Shirokane, Minato-ku, Tokyo, 108-8641 Japan; 2 Division of Clinical Laboratory, Sanritsu Zelkova Veterinary Laboratory, Tokyo, Japan; 3 Department of Laboratory Medicine, Kangdong Sacred Heart Hospital, Hallym University College of Medicine, Seoul, Republic of Korea

**Keywords:** Genetic organization, M protein trans-acting positive regulator (Mga), M-like protein (SCM), *Streptococcus canis*

## Abstract

**Objective:**

The purpose of this study was to identify the M protein trans-acting positive regulator (Mga) orthologue and its adjacent M-like protein (SCM) alleles in *Streptococcus canis*.

**Results:**

Using the 39 SCM allele isolates and polymerase chain reaction-based amplification and sequencing, we obtained the deduced Mga amino acid (AA) sequences. The 22 Mga sequences in whole-genome sequences were obtained by searching the National Collection of Type Cultures 12,191(T) Mga sequence into the database. The percentage identity to the type-strain Mga sequence was examined along with its size. The presence of the Mga-specific motifs was confirmed. Of the 62 strains, we identified 59 Mga sequences with an AA size of 509 (except for four different sizes). Percentage identity ranged from 96.66 to 100% with the confirmed Mga-specific motifs and diverse SCM allele populations. Our findings support the presence of an Mga orthologue and diverse SCM allele populations.

## Introduction

In 1986, Devriese et al. [1] designated a species of Lancefield carbohydrate antigenicity group G streptococci from animals as *Streptococcus canis*. On sheep blood agar plates, this microorganism forms large, smooth, gray/white-colored colonies with β-hemolysis. In healthy dogs, *S*. *canis* constitutes part of the resident microflora of the oropharynx, skin, urogenital tract, and anus [2]. This bacterium is an emerging pathogen causing self-limiting dermatitis among companion animals (i.e., dogs or cats) [3]. However, *S*. *canis* infection can occasionally result in severe diseases in dogs and cats, including arthritis, streptococcal toxic shock syndrome, necrotizing fasciitis, septicemia, and pneumonia [4, 5]. We have also reported a case of severe soft tissue infection with septic shock caused by *S. canis* in a miniature dachshund [6]. Additionally, *S*. *canis* can infect humans who have been in close contact with companion animals and cause either local or systemic diseases [7]. This species was recovered from two Japanese patients with bacteremia who were in deep contact with or bitten by pet dogs [8, 9]. Periprosthetic joint infection with *S. canis* has been described in a man undergoing elective primary total hip arthroplasty [10]; a pet dog had frequently licked his legs. Many Japanese individuals keep dogs or cats in their homes. Moreover, medical institutes and nursing homes are introducing animal-assisted therapy as a mental health service for hospital patients and elderly individuals. Companion animals and humans are living closely. Thus, it is important for veterinary and medical doctors to be aware of the possibility of *S*. *canis*-related zoonotic infections being underdiagnosed.

The *S. canis* M-like protein (SCM), which is encoded by the *scm* gene, can bind to plasminogen and immunoglobulin G and confers antiphagocytic properties [11]. We performed polymerase chain reaction (PCR)-based amplification of *scm* (with amplicon sizes of 1,700–2,100 bp) and conducted direct sequencing [12, 13]. We constructed an unrooted phylogenetic tree of the deduced amino acid (AA) sequences using the neighbor-joining method. SCM allele typing was performed based on different/similar positions using variable/conserved AA sequences in the phylogenetic tree. Allele types were classified into two groups: group I, with relatively similar sequences (consisting of allele types 1–9) [14] and group II, with diverse sequences (consisting of allele types 10–15) [12]. The typing in group I was performed based on variable AA sequences with signal peptide types at the amino terminus [14].

*Streptococcus pyogenes*, a virulent human pathogen exhibiting carbohydrate group A, also possesses an antiphagocytic M protein (encoded by the *emm* gene). The M protein’s trans-acting positive regulator, also known as the multiple gene activator (Mga), is a DNA-binding transcriptional activator protein. Mga can enhance the expression of multiple genes, such as *emm*, *scpA*, which encodes C5a peptidase, and *mga* itself, implying that it is an autoregulator [15]. The *mga*-*emm*-*scpA* genes are closely linked and arranged in tandem, and these genes are referred to as the ‘*mga* regulon’. Helix-turn-helix (HTH) AA secondary structures have DNA-binding activities. The amino-terminus of Mga contains four potential HTH DNA-binding domains (HTH1–HTH4); two of these domains, HTH3 and HTH4, are needed for direct activation of the ‘*mga* regulon’ in vivo [16]. Furthermore, it has been established that the conserved Mga domain 1 (CMD-1) likely contributes to the protein stability with (auto)activation [17]. Thus, HTH3/HTH4 and CMD-1 are targeted for Mga functional analysis.

*Streptococcus dysgalactiae* subsp. *equisimilis* (SDSE) and subsp. *dysgalactiae*, *S. equi*, *S. gordonii*, *S. mitis*, and *S*. *pneumoniae* also contain Mga ortholgues [17]. However, there are very few descriptions regarding the genetic organization of the *scm* gene region, which is similar with the *mga*–*emm*–*scpA* linkage. Thus, the purpose of this study was to examine the presence of an Mga orthologue and its related SCM alleles in *S. canis*.

## Methods

### Comparison of genomic structures from S. pyogenes and S. canis strains

We performed the comparison of genomic structures from *S. pyogenes* strain JRS4 and *S. canis* National Collection of Type Cultures (NCTC) 12,191(T). Additionally, the comparison of genomic structures from other *S. pyogenes* strains and other *S. canis* strains was carried out. Genomic structures were constructed based on the whole-genome sequence (WGS) graphics specified in the GenBank descriptions of the National Center for Biotechnology Information (NCBI) database.

### PCR-based amplification and direct sequencing of mga gene

**Fig. 1:**
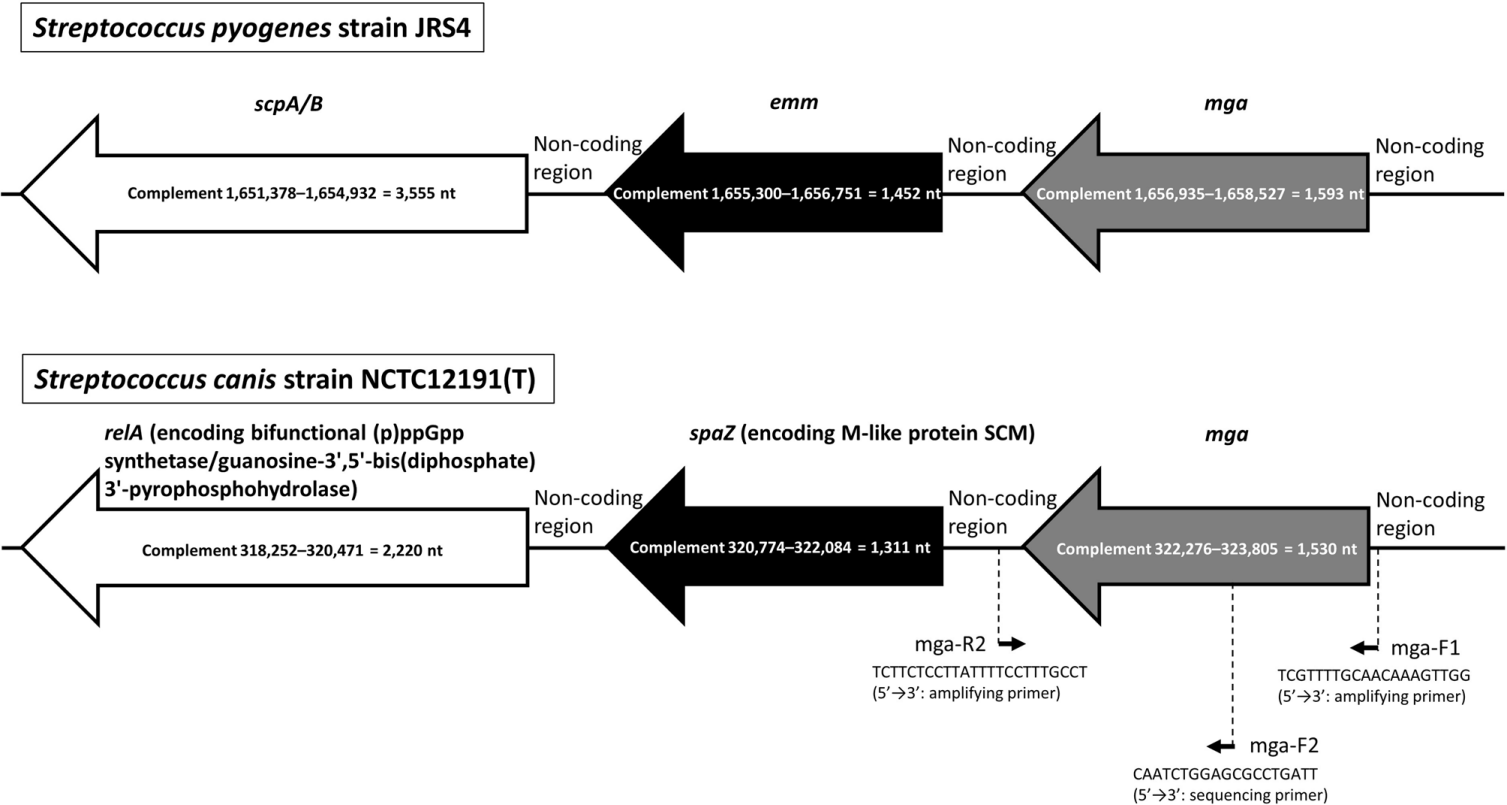
Two genomic structures from *Streptococcus pyogenes* strain JRS4 and *Streptococcus canis* strain National Collection of Type Cultures (NCTC) 12,191(T). These structures were constructed based on the whole-genome sequence (WGS) graphics specified in the GenBank descriptions (accession numbers CP011414.1 and LR134293.1) of the National Center for Biotechnology Information database

We enrolled *S. canis* isolates collected during the three previous study periods in 2015 (*n* = 17), 2017 (*n* = 6), and 2021 (*n* = 16) (Table 1) [18–20]. The isolates were identified based on the 16 S sequencing results. The corresponding animal information regarding sex and year-age is shown in Table 1. The thirty-nine isolates contained the determined SCM alleles (including the truncated variants) (Table 1). Streptococci genomic DNA was extracted by suspension in 10 mM Tris-1 mM EDTA (pH 8.0), followed by boiling at 97 °C for 10 min and a brief microfuge step after the boiling lysis [21]. Two amplifying primers and one sequencing primer (mga-F1, mga-R2, and mga-F2 shown in Fig. 1) were designed based on the WGS of *S. canis* NCTC 12,191(T) using the web-based application Primer3Plus [22]. NCTC 12,191(T)-origin DNA was used as a positive control, and DNase/RNase/protease-free water was used as a negative control in each PCR assay. PCR was performed with an initial denaturation step at 94 °C for 1 min, followed by 30 cycles (consisting of denaturation at 94 °C for 1 min, annealing at 50 °C for 1 min, and extension at 72 °C for 2 min), and a final extension step at 72 °C for 10 min. PCR products with the expected amplicon size (1801 bp) were separated using 1.5% agarose gel electrophoresis in Tris-acetate-EDTA buffer. Direct sequencing after amplicon purification by QIAquick PCR Purification Kit (Qiagen, Tokyo, Japan) was conducted on the Applied Biosystems 3730xl DNA Analyzer with the BigDye Terminator V3.1 (Thermo Fisher Scientific, Waltham, MA, USA). We obtained the coding DNA sequences and deduced the AA sequences.

**Table 1:** M protein trans-acting positive regulator (Mga) sequence and its adjacent M-like protein (SCM) allele of *Streptococcus canis*

Strain	Host (sex, year-age)	Year	Geographic location	Isolation source	GenBank accession no. of mga (size)	% identity to type strain Mga AA sequence (size)	AA sequence at positions 11–16	AA sequence at positions 54–73*	AA sequence at positions 108–127*	SCM allele (allele group)**
NCTC 12,191(T)	Bovine	Unknown	Unknown	Mastitis	LR134293.1 (1,530 bp)	100% (509)	**Q**QWRE**L**	LQFMESLGRIT**YK**DSYLSID	LEDLAEALFIS**LS**TLKRLIE	Allele 1 (group I)
NCTC 6198	Animal	Unknown	United Kingdom	Unknown	CABEII010000002.1 (1,530 bp)	99.21% (509)	**Q**QWRE**L**	LQFMESLGRIT**YK**DSYLSID	LEDLAEALFIS**LS**TLKRLIE	Allele 1 (group I)
FSL Z3-227	Cow	1999	USA: New York	Milk	AIDX01000001.2 (1,530 bp)	100% (509)	**Q**QWRE**L**	LQFMESLGRIT**YK**DSYLSID	LEDLAEALFIS**LS**TLKRLIE	Allele 1 (group I)
FMV2238	Dog	2002	Portugal: Lisbon	Ear	UXEP01000025.1 (1,530 bp)	97.25% (509)	**Q**QWRE**L**	LQFMESLGRIT**YK**DSYLSID	LEDLAEALFIS**LS**TLKRLIE	Allele 15 (group II)
G361	Human	2006	Germany: Lower Saxony	Vagina	NMRV01000013.1 (1,530 bp)	98.62% (509)	**Q**QWRE**L**	LQFMESLGRIT**YK**DSYLSID	LEDLAEALFIS**LS**TLKRLIE	Allele 4 (group I)
OT1	Human	2012	Japan: Gifu	Blood	BJOW01000005.1 (1,530 bp)	99.02% (509)	**Q**QWRE**L**	LQFMESLGRIT**YK**DSYLSID	LEDLAEALFIS**LS**TLKRLIE	Allele 1 (group I)
cVljOFJyGN_bin.30.MAG	Human	2013	USA	Skin	CALTTA010000009.1 (1,530 bp)	99.21% (509)	**Q**QWRE**L**	LQFMESLGRIT**YK**DSYLSID at positions 64–83	LEDLAEALFIS**LS**TLKRLIE at positions 118–137	Allele 10 (group II)
SA2	Dog (F, 5)	2015	Japan: Aichi	Urine	LC777209 (1,530 bp)	99.41% (509)	**Q**QWRE**L**	LQFMESLGRIT**HK**DSYLSID	LEDLAEALFIS**LS**TLKRLIE	Allele 11 (group II)
SA3	Dog (M, 9)	2015	Japan: Tokyo	Ear wax	LC777210 (1,530 bp)	97.84% (509)	**Q**QWRE**L**	LQFMESLGRIT**HK**DSYLSID	LEDLAEALFIS**LS**TLKRLIE	Allele 2 (group I)
SA5	Cat (F, 7)	2015	Japan: Chiba	Blood	LC777211 (1,530 bp)	98.43% (509)	**Q**QWRE**L**	LQFMESLGRIT**YK**DSYLSID	LEDLAEALFIS**LS**TLKRLIE	Allele 8 (group I)
SA8	Dog (M, 6)	2015	Japan: Aichi	Urine	LC777212 (1,530 bp)	98.82% (509)	**Q**QWRE**L**	LQFMESLGRIT**YK**DSYLSID	LEDLAEALFIS**LS**TLKRLIE	Allele 2 (group I)
SA10	Dog (M, 14)	2015	Japan: Ibaraki	Ear discharge	LC777213 (1,530 bp)	97.25% (509)	**Q**QWRE**L**	LQFMESLGRIT**YK**DSYLSID	LEDLAEALFIS**LS**TLKRLIE	Allele 15 (group II)
SA12	Dog (unknown, unknown)	2015	Japan: Wakayama	Ear discharge	LC777214 (1,530 bp)	97.84% (509)	**Q**QWRE**L**	LQFMESLGRIT**YK**DSYLSID	LEDLAEALFIS**LS**TLKRLIE	Allele 3 (group I)
SA16	Dog (M, 17)	2015	Japan: Aichi	Pus	LC777215 (1,530 bp)	99.21% (509)	**Q**QWRE**L**	LQFMESLGRIT**YK**DSYLSID	LEDLAEALFIS**LS**TLKRLIE	Allele 1 (group I)
SA18	Cat (M, 1)	2015	Japan: Ibaraki	Nasal discharge	LC777216 (1,530 bp)	97.45% (509)	**Q**QWRE**L**	LQFMESLGRIT**YK**DSYLSID	LEDLAEALFIS**LS**TLKRLIE	Allele 13 (group II)
SA25	Dog (M, unknown)	2015	Japan: Chiba	Ear wax	LC777217 (1,530 bp)	99.61% (509)	**Q**QWRE**L**	LQFMESLGRIT**YK**DSYLSID	LEDLAEALFIS**LS**TLKRLIE	Allele 10 (group II)
SA26	Dog (F, 9)	2015	Japan: Chiba	Oral cavity	LC777218 (1,530 bp)	97.25% (509)	**Q**QWRE**L**	LQFMESLGRIT**YK**DSYLSID	LEDLAEALFIS**LS**TLKRLIE	Allele 15 (group II)
SA32	Dog (F, 10)	2015	Japan: Shizuoka	Ear discharge	LC777219 (1,530 bp)	99.02% (509)	**Q**QWRE**L**	LQFMESLGRIT**YK**DSYLSID	LEDLAEALFIS**LS**TLKRLIE	***A truncated variant***
SA34	Dog (M, 10)	2015	Japan: Osaka	Ear discharge	LC777220 (1,530 bp)	99.61% (509)	**Q**QWRE**L**	LQFMESLGRIT**YK**DSYLSID	LEDLAEALFIS**LS**TLKRLIE	Allele 10 (group II)
SA57	Dog (M, 8)	2015	Japan: Niigata	Pus	LC777221 (1,530 bp)	99.61% (509)	**Q**QWRE**L**	LQFMESLGRIT**YK**DSYLSID	LEDLAEALFIS**LS**TLKRLIE	Allele 10 (group II)
SA68	Cat (M, 7)	2015	Japan: Okinawa	Urine	LC777222 (1,530 bp)	100% (509)	**Q**QWRE**L**	LQFMESLGRIT**YK**DSYLSID	LEDLAEALFIS**LS**TLKRLIE	Allele 1 (group I)
SA69	Dog (M, unknown)	2015	Japan: Fukui	Ear discharge	LC777223 (1,530 bp)	96.66% (509): minimum % identity	**Q**QWRE**L**	LQFMESLGRIT**YK**DSYLSID	LEDLAEALFIS**LS**TLKRLIE	Allele 12 (group II)
SA71	Cat (M, 9)	2015	Japan: Aichi	Ear discharge	LC777224 (1,530 bp)	97.45% (509)	**Q**QWRE**L**	LQFMESLGRIT**YK**DSYLSID	LEDLAEALFIS**LS**TLKRLIE	Allele 13 (group II)
SA72	Dog (M, 11)	2015	Japan: Chiba	Pus	LC777225 (1,530 bp)	98.82% (509)	**Q**QWRE**L**	LQFMESLGRIT**YK**DSYLSID	LEDLAEALFIS**LS**TLKRLIE	Allele 4 (group I)
TA4	Human	2016	Japan: Tokyo	Blood	BEWZ01000005.1 (1,530 bp)	99.02% (509)	**Q**QWRE**L**	LQFMESLGRIT**YK**DSYLSID	LEDLAEALFIS**LS**TLKRLIE	Allele 1 (group I)
B700072	Dog	2017	United Kingdom	Cornea	LR590625.1 (1,530 bp)	98.62% (509)	**Q**QWRE**L**	LQFMESLGRIT**YK**DSYLSID	LEDLAEALFIS**LS**TLKRLIK	Allele 4 (group I)
FU1	Cat (M, unknown)	2017	Japan: Chiba	Pus	BLIS01000014.1 (1,530 bp)	99.41% (509)	**Q**QWRE**L**	LQFMESLGRIT**YK**DSYLSID	LEDLAEALFIS**LS**TLKRLIE	Allele 11 (group II)
FU6	Cat (M, 6)	2017	Japan: Okayama	Pus	BLIT01000007.1 (1,530 bp)	99.21% (509)	**Q**QWRE**L**	LQFMESLGRIT**YK**DSYLSID	LEDLAEALFIS**LS**TLKRLIE	Allele 1 (group I)
FU22	Dog (F, 12)	2017	Japan: Tokyo	Ear discharge	LC777226 (1,530 bp)	98.43% (509)	**Q**QWRE**L**	LQFMESLGRIT**YK**DSYLSID	LEDLAEALFIS**LS**TLKRLIE	Allele 2 (group I)
FU25	Dog (M, 9)	2017	Japan: Chiba	Ear discharge	LC777227 (1,530 bp)	96.66% (509): minimum % identity	**Q**QWRE**L**	LQFMESLGRIT**YK**DSYLSID	LEDLAEALFIS**LS**TLKRLIE	Allele 12 (group II)
FU29	Dog (F, 6)	2017	Japan: Kanagawa	Vagina	BLKN01000014.1 (1,530 bp)	98.62% (509)	**Q**QWRE**L**	LQFMESLGRIT**YK**DSYLSID	LEDLAEALFIS**LS**TLKRLIE	Allele 5 (group I)
FU53	Cat (F, unknown)	2017	Japan: Chiba	Nasal cavity	BLKO01000011.1 (1,530 bp)	99.61% (509)	**Q**QWRE**L**	LQFMESLGRIT**YK**DSYLSID	LEDLAEALFIS**LS**TLKRLIE	Allele 10 (group II)
FU64	Dog (M, 13)	2017	Japan: Nagasaki	Ear discharge	LC777228 (1,530 bp)	97.64% (509)	**Q**QWRE**L**	LQFMESLGRIT**YK**DSYLSID	LEDLAEALFIS**LS**TLKRLIE	Allele 3 (group I)
FU69	Cat (M, unknown)	2017	Japan: Saitama	Pus	LC777229 (1,530 bp)	97.25% (509)	**Q**QWRE**L**	LQFMESLGRIT**YK**DSYLSID	LEDLAEALFIS**LS**TLKRLIE	Allele 13 (group II)
FU70	Dog (M, 2)	2017	Japan: Tokyo	Conjunctiva	LC777230 (1,530 bp)	99.21% (509)	**Q**QWRE**L**	LQFMESLGRIT**YK**DSYLSID	LEDLAEALFIS**LS**TLKRLIE	Allele 7 (group I)
FU93	Dog (F, 9)	2017	Japan: Chiba	Pus	BLKP01000024.1 (1,530 bp)	99.61% (509)	**Q**QWRE**L**	LQFMESLGRIT**YK**DSYLSID	LEDLAEALFIS**LS**TLKRLIE	Allele 10 (group II)
FU97	Dog (M, 11)	2017	Japan: Okayama	Pus	BLKQ01000009.1 (1,530 bp)	98.82% (509)	**Q**QWRE**L**	LQFMESLGRIT**YK**DSYLSID	LEDLAEALFIS**LS**TLKRLIE	Allele 4 (group I)
FU100	Cat (F, 12)	2017	Japan: Chiba	Pus	LC777231 (1,530 bp)	99.41% (509)	**Q**QWRE**L**	LQFMESLGRIT**HK**DSYLSID	LEDLAEALFIS**LS**TLKRLIE	Allele 14 (group II)
FU129	Dog (M, 9)	2017	Japan: Niigata	Pus	BLIU01000007.1 (1,530 bp)	99.41% (509)	**Q**QWRE**L**	LQFMESLGRIT**YK**DSYLSID	LEDLAEALFIS**LS**TLKRLIE	Allele 10 (group II)
HL_77_1	Dog	2018	Korea: Seoul	Ear	CP053792.1 (1,530 bp)	97.05% (509)	**Q**QWRE**L**	LQFMESLGRIT**YK**DSYLSID	LEDLAEALFIS**LS**TLKRLIE	Allele 15 (group II)
HL_77_2	Dog	2018	Korea: Seoul	Ear	CP053790.1 (1,530 bp)	98.23% (509)	**Q**QWRE**L**	LQFMESLGRIT**YK**DSYLSID	LEDLAEALFIS**LS**TLKRLIE	Allele 2 (group I)
HL_98_2	Dog	2018	Korea: Seoul	Nasal cavity	CP053789.1 (1,530 bp)	99.61% (509)	**Q**QWRE**L**	LQFMESLGRIT**YK**DSYLSID	LEDLAEALFIS**LS**TLKRLIE	Allele 10 (group II)
HL_100	Dog	2018	Korea: Seoul	Urine	CP046521.1 (1,530 bp)	98.62% (509)	**Q**QWRE**L**	LQFMESLGRIT**YK**DSYLSID	LEDLAEALFIS**LS**TLKRLIE	Allele 4 (group I)
FU149	Dog (M, 13)	2019	Japan: Chiba	Blood	BLRR01000038.1 (1,530 bp)	99.41% (509)	**Q**QWRE**L**	LQFMESLGRIT**YK**DSYLSID	LEDLAEALFIS**LS**TLKRLIE	Allele 1 (group I)
IMT49926	Dog	2020	Germany: Berlin	Blood	JARLUA010000012.1 (1,590 bp)	97.45% (529)	**Q**QWRE**L** at positions 31–36	LQFMESLGRIT**YK**DSYLSID at positions 74–93	LEDLAEALFIS**LS**TLKRLIE at positions 128–147	Allele 13 (group II)
KU4	Dog (M, 10)	2021	Japan: Tokyo	Eye discharge	LC777232 (1,530 bp)	97.45% (509)	**Q**QWRE**L**	LQFMESLGRIT**YK**DSYLSID	LEDLAEALFIS**LS**TLKRLIE	Allele 13 (group II)
KU10	Dog (M, 13)	2021	Japan: Tokyo	Fluid in tympanic cavity	LC777233 (1,530 bp)	98.62% (509)	**Q**QWRE**L**	LQFMESLGRIT**YK**DSYLSID	LEDLAEALFIS**LS**TLKRLIE	Allele 6 (group I)
KU16	Cat (M, unknown)	2021	Japan: Chiba	Pus	LC777234 (1,530 bp)	97.45% (509)	**Q**QWRE**L**	LQFMESLGRIT**YK**DSYLSID	LEDLAEALFIS**LS**TLKRLIE	Allele 13 (group II)
KU29	Cat (F, 12)	2021	Japan: Saitama	Pus	LC777235 (1,530 bp)	98.82% (509)	**Q**QWRE**L**	LQFMESLGRIT**YK**DSYLSID	LEDLAEALFIS**LS**TLKRLIE	***A truncated variant***
KU31	Dog (F, unknown)	2021	Japan: Tokyo	Pus	LC777236 (1,531 bp)	***A truncated variant: 98.41% (126)***	**Q**QWRE**L**	LQFMESLGRIT**YK**DSYLSID	LEDLAEALFIS**LS**TLKTLD	Allele 14 (group II)
KU41	Cat (F, 14)	2021	Japan: Tokyo	Pus	LC777237 (1,530 bp)	98.23% (509)	**Q**QWRE**L**	LQFMESLGRIT**YK**DSYLSID	LEDLAEALFIS**LS**TLKRLIE	***A truncated variant***
KU42	Dog (M, 3)	2021	Japan: Saitama	Ear discharge	LC777238 (1,530 bp)	98.04% (509)	**Q**QWRE**L**	LQFMESLGRIT**YK**DSYLSID	LEDLAEALFIS**LS**TLKRLIE	Allele 2 (group I)
KU57	Dog (M, 6)	2021	Japan: Tokyo	Cornea	LC777239 (1,530 bp)	98.04% (509)	**Q**QWRE**L**	LQFMESLGRIT**HK**DSYLSID	LEDLAEALFIS**LS**TLKRLIE	Allele 2 (group I)
KU59	Dog (F, 13)	2021	Japan: Tokyo	Uterus content	LC777240 (1,530 bp)	97.25% (509)	**Q**QWRE**L**	LQFMESLGRIT**YK**DSYLSID	LEDLAEALFIS**LS**TLKRLIE	Allele 15 (group II)
KU69	Dog (F, 9)	2021	Japan: Tokyo	Fluid in ear canal	LC777241 (1,530 bp)	98.82% (509)	**Q**QWRE**L**	LQFMESLGRIT**YK**DSYLSID	LEDLAEALFIS**LS**TLKRLIE	Allele 9 (group I)
KU72	Dog (F, 9)	2021	Japan: Chiba	Vaginal discharge	LC777242 (1,530 bp)	97.84% (509)	**Q**QWRE**L**	LQFMESLGRIT**YK**DSYLSID	LEDLAEALFIS**LS**TLKRLIE	Allele 3 (group I)
KU82	Dog (F, 15)	2021	Japan: Tokyo	Ear discharge	LC777243 (1,530 bp)	99.41% (509)	**Q**QWRE**L**	LQFMESLGRIT**YK**DSYLSID	LEDLAEALFIS**LS**TLKRLIE	Allele 6 (group I)
KU84	Dog (F, 12)	2021	Japan: Tokyo	Uterus pus	LC777244 (1,530 bp)	99.02% (509)	**Q**QWRE**L**	LQFMESLGRIT**YK**DSYLSID	LEDLAEALFIS**LS**TLKRLIE	Allele 9 (group I)
KU96	Cat (F, 13)	2021	Japan: Tokyo	Pus	LC777245 (1,529 bp)	***A truncated variant: 98.25% (400)***	**Q**QWRE**L**	LQFMESLGRIT**YK**DSYLSID	LEDLAEALFIS**LS**TLKRLIE	Allele 8 (group I)
KU106	Dog (M, 10)	2021	Japan: Aichi	Urine	LC777246 (1,530 bp)	96.86% (509)	**Q**QWRE**L**	LQFMESLGRIT**YK**DSYLSID	LEDLAEALFIS**LS**TLKRLIE	Allele 12 (group II)
KU109	Cat (F, 1)	2021	Japan: Iwate	Pus	LC777247 (1,530 bp)	***A truncated variant: 100% (10)***	Not available	Not available	Not available	Allele 8 (group I)
bin-133	Cat	2021/2022	USA: California	Anal gland secretions	JASCAB010000028.1 (1,530 bp)	99.41% (509)	**Q**QWRE**L**	LQFMESLGRIT**YK**DSYLSID	LEDLAEALFIS**LS**TLKRLIE	Allele 6 (group I)

AA, amino acid; NCTC, National Collection of Type Cultures; M, male; F, female. Gray shading shows whole-genome sequences and their related information. *Two flanking AAs likely involved in protein stability and two AAs within each ‘recognition’ helix expected to bind to DNA are underlined in bold letters

The frameshift sites were verified by inspection of chromatograms at the corresponding positions. Truncated variants are shown in italic and bold letters

AA sequences at positions 10–15, 53–72, and 107–126 of *S. pyogenes* strain JRS4 Mga (GenBank accession no. CP011414.1) were **Q**QWRE**L**, MQFMKEVGGIT**YK**NGYITIW, and LEELAEELFVS**LS**TLKRLIK, respectively. **M-like protein allele typing was conducted based on our previous typing methods

We found no significant associations between SCM group I and host (humans) or isolation source (sterile samples) using a two-sided Fisher’s exact test

### WGS-based detection of mga and scm genes

We retrieved WGSs from *S. canis* strains (*n* = 22), along with the WGS of NCTC 12,191(T) (gray shading in Table 1), which were deposited in the NCBI database (updated August 1, 2023) for this retrospective study. The Japanese animal information regarding sex and year-age is shown in Table 1. Putative Mga-related nucleotide/AA sequences were obtained by inserting NCTC 12,191(T) Mga sequence into the NCBI Nucleotide/Protein Basic Local Alignment Search Tool [23]. We also retrieved SCM nucleotide/AA sequences adjacent to Mga. Allele typing was performed based on two previous typing methods, and the alleles were classified into the groups I–II [12, 14].

### Determination of Mga-specific AA motifs and percent identity to Mga AA sequence in the type-strain

We examined the presence of Mga-specific AA motifs in *S*. *canis* as compared to those of *S. pyogenes* [16, 17]. The percentage identity to Mga AA sequence in NCTC 12,191(T) was examined, along with its AA size in each strain.

All analyses were conducted at the Graduate School of Infection Control Sciences and Ōmura Satoshi Memorial Institute, Kitasato University.

## Results

### Comparison of genomic structures from S. pyogenes and S. canis strains

Figure 1 shows two genomic structures from *S. pyogenes* JRS4 and *S. canis* type-strain. These structures were made based on the WGS graphics specified in the GenBank descriptions (accession numbers CP011414.1 and LR134293.1). The genetic organization between the *mga*-*emm* locus is consistent with that between the *mga*-*scm* (*spaZ*) locus. In contrast, the downstream gene arrangements were different. The *scpA/B* is located at 367 nucleotides downstream of *emm* in *S*. *pyogenes*, whereas the *relA* (encoding bifunctional (p)ppGpp synthetase/guanosine-3’,5’-bis(diphosphate) 3’-pyrophosphohydrolase) is located at 302 nucleotides downstream of *scm* (*spaZ*) in *S*. *canis*. In other *S. pyogenes* strains (NCTC 8198/Culture Collection University of Gothenburg 4207/1085), there was the organization between the *mga*-*emm* locus and the different downstream arrangement (including *scpA/B*) of *emm*. For example, these three strains had the *mga*-*emm*-gene (encoding YSIRK-type signal peptide-containg protein)-sic./gene (encoding lysis inhibitor protein)-gene (encoding IS1182 family transposase)-*scpA/B* arrangement. In other *S. canis* strains (NCTC 6198/OT1/TA4), there was the organization between the *mga*-*scm* (*spaZ*)-*relA* locus. Furthermore, we found the *relA* possession in *S. pyogenes* strains and the *scp* possession in *S. canis* strains. The *relA* was shown to be located at distant position from the *mga*-*emm* locus and the *scp* was shown to be located at distant position from the *mga*-*scm* locus.

### Background information about enrolled strains

Table 1 lists the strain background information recorded in our previous investigations (2015–2017–2021) or in the NCBI database. The enrolled strains were from animals (*n* = 58) and humans (*n* = 4); the collection years were from 1999 to 2021/2022; the geographic location included forty-nine isolates from Japan and 12 overseas strains; and the isolation sources constituted seven invasive strains (from blood and uterus) and fifty-four noninvasive strains (mainly from ear, pus, urogenital tract, eye, and nose).

### Characterization of an Mga orthologue and SCM alleles

**Fig. 2:**
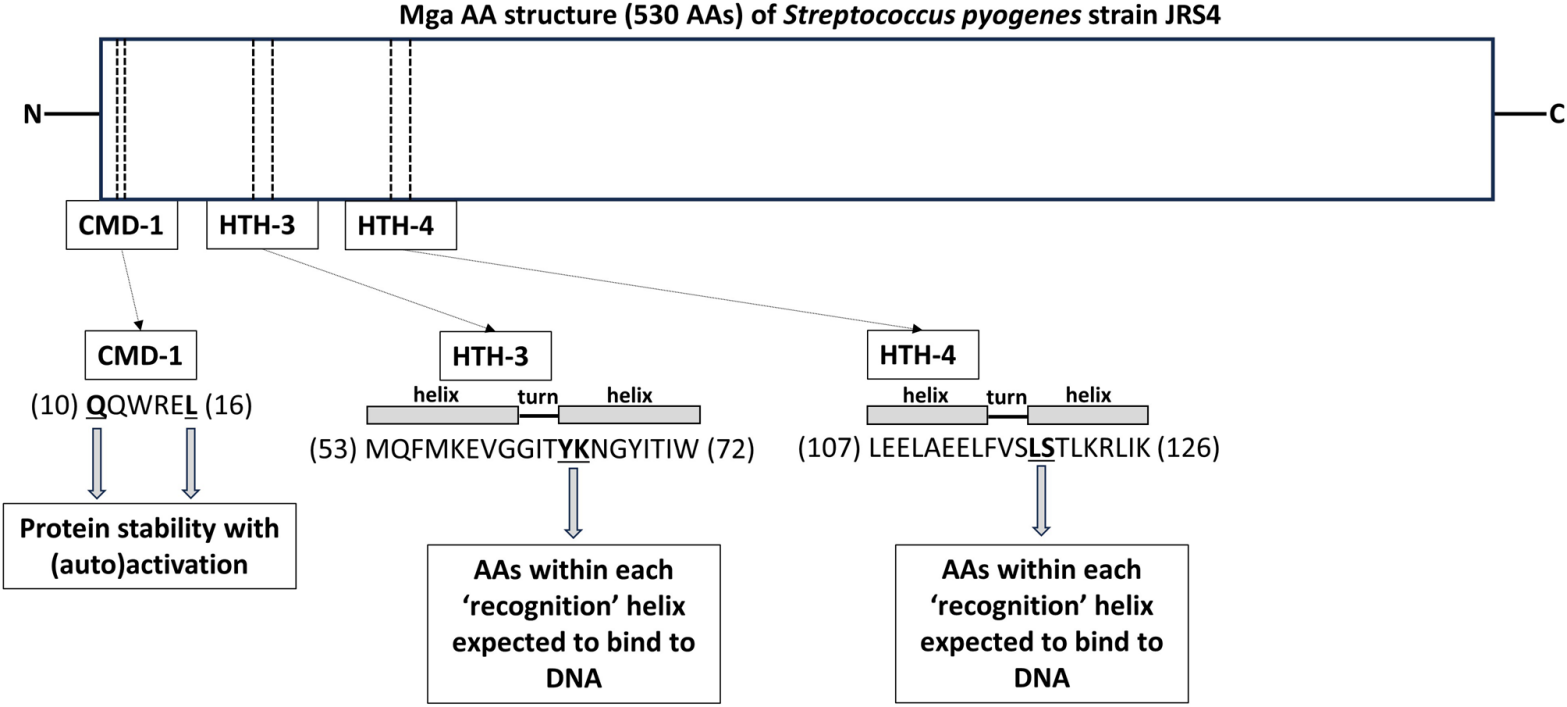
Multiple gene activator (Mga) amino acid (AA) structure (530 AAs) of *Streptococcus pyogenes* strain JRS4 is shown on the upper side. Potential three functional domains are conserved Mga domain 1 (CMD-1) and helix-turn-helix (HTH) DNA-binding domain 3–4 (HTH3–HTH4) that are located at the amino terminus [15, 16]. AA residues composing the three functional domains are shown on the lower side. AA positions are indicated in parentheses. HTH3/HTH4 with **YK** and **LS** motifs and CMD-1 with two flanking (**Q**–**L**) are targeted for Mga functional analysis

The detailed results regarding Mga nucleotide/AA sizes, percentage identity to the type-strain Mga sequence, along with AA sequences at CMD-1 and HTH3/HTH4 domains in each strain are shown in Table 1. We observed *mga* nucleotide size of 1,530 bp (except for 1,590 bp and 1,529 and 1,531 bp resulting in two truncated variants) and Mga AA size of 509 (except for 529 AA and 10–126–400 AAs of three truncated variants). The percentage identity ranged from a minimum of 96.66% to a maximum of 100%. We found the presence of CMD-1 (including two flanking AAs: **Q**–**L**) and two HTH3/HTH4 domains (containing **YK** and **LS** motifs) at the amino-terminus to assess the potential Mga orthologous structure associated with its function, because the AA sequences at positions 10–15, 53–72, and 107–126 of *S. pyogenes* strain JRS4 Mga (530 AAs) were **Q**QWRE**L**, MQFMKEVGGIT**YK**NGYITIW, and LEELAEELFVS**LS**TLKRLIK, respectively (Fig. 2). Almost all the strains (except for a truncated variant strain KU109 shown in Table 1) had the CMD-1 (including two flanking AAs: **Q**–**L**) at AA positions 11–16 or 31–36. Additionally, almost all the strains (except for the truncated variant KU109) possessed the HTH3 domain (containing **YK**/**HK** motifs) at positions 54–73, 64–83, or 74–93. Furthermore, almost all the strains (except for the truncated variant KU109) contained the HTH4 domain (containing **LS** motif) at positions 108–127, 118–137, or 128–147. Thus, we confirmed the potential Mga orthologous structure associated with its function among the registered strains. In contrast, we observed the diverse SCM allele populations consisting of groups I (*n* = 33) and II (*n* = 26), along with three truncated variants. Group I included alleles 1–9, whereas group II included alleles 10–15.

## Discussion

Group C SDSE, which is closely related to *S. canis*, has an orthologous gene (*mgc*), a multigene regulator. Mgc (513 AA) in SDSE strain H46A was 51.3% identical to Mga in *S. pyogenes* strain D471 [24]. The phylogenetic analysis indicated that Mgc in SDSE constituted a distinct cluster separated from Mga in *S. pyogenes* [24]. It seems likely that the SDSE/*S. canis mgc*/*mga* and *S. pyogenes mga* have undergone a considerable period of independent evolutionary development.

We searched for related articles by entering the keywords “streptococcus canis, transcriptional regulator,” “streptococcus canis, multiple gene activator,” and “streptococcus canis, DNA-binding” in the PubMed [25]. However, there appear to be no adequate hits in related manuscripts (as of January 11, 2024). To the best of our knowledge, this is the first report of a homologous sequence of Mga and its adjacent diverse SCM alleles in *S. canis*, suggesting its operon, which is similar with the *S. pyogenes* ‘*mga* regulon’. Based on the diversity, we further should establish the SCM allele typing for molecular epidemiological approaches. Two *mga* alleles (*mga-1* and *mga-2*) are found within *S. pyogenes* based on their ability to bind to an oligonucleotide probe [26] and are associated with different genetic patterns at *mga* locus and different tissue tropisms [27]. Therefore, it is important to carry out sequential analysis among additional *S. canis* strains to monitor the development of MGA alleles in our future observations.

## Limitations

We need to further determine whether this molecule has the functional ability to bind to *scm*, *mga*, and other genetic regions including their promoter sequences and to activate their transcription by in vitro/in vivo experiments.

## Data Availability

Sequence data that support the findings of this study have been deposited in the National Center for Biotechnology Information, US. Table 1 lists the corresponding GenBank nucleotide accession numbers of mga gene.

## Abbreviations

AA: amino acid
BLAST: Basic Local Alignment Search Tool
CMD: conserved Mga domain
HTH: helix-turn-helix
Mga: multiple gene activator
Mgc: multigene regulator C
NCBI: National Center for Biotechnology Information
NCTC: National Collection of Type Cultures
PCR: polymerase chain reaction
SCM: *S. canis* M-like protein
SDSE: *Streptococcus dysgalactiae* subsp. *Equisimilis*
WGS: whole-genome sequence

## Declarations

### Author information

TT and J-SK research foci are to characterize the virulence factors, epidemiological data, antimicrobial resistance patterns in Gram-positive cocci (i.e., genus *Streptococcus* and genus *Staphylococcus*) isolated from human patients and diseased companion animals.
